# Genomic characteristics of a novel emerging PRRSV branch in sublineage 8.7 in China

**DOI:** 10.3389/fmicb.2023.1186322

**Published:** 2023-05-31

**Authors:** Wansheng Li, Chao Li, Zhenyang Guo, Hu Xu, Bangjun Gong, Qi Sun, Jing Zhao, Lirun Xiang, Chaoliang Leng, Jinmei Peng, Guohui Zhou, Yandong Tang, Huairan Liu, Tongqing An, Xue-Hui Cai, Zhi-Jun Tian, Qian Wang, Hongliang Zhang

**Affiliations:** ^1^State Key Laboratory for Animal Disease Control and Prevention, Harbin Veterinary Research Institute, Chinese Academy of Agricultural Sciences, Harbin, China; ^2^Henan Key Laboratory of Insect Biology in Funiu Mountain, Henan Provincial Engineering Laboratory of Insects Bio-Reactor, China-UK-NYNU-RRes Joint Laboratory of Insect Biology, Nanyang Normal University, Nanyang, China

**Keywords:** PRRSV, new branch, sublineage 8.7, 1 + 8 + 1, genomic characteristics

## Abstract

Porcine reproductive and respiratory syndrome virus (PRRSV) has caused serious economic losses to the pig industry worldwide. During the continuous monitoring of PRRSV, a new PRRSV strain type with novel characteristics was first identified in three different regions of Shandong Province. These strains presented a novel deletion pattern (1 + 8 + 1) in the NSP2 region and belonged to a new branch in sublineage 8.7 based on the ORF5 gene phylogenetic tree. To further study the genomic characteristics of the new-branch PRRSV, we selected a sample from each of the three farms for whole-genome sequencing and sequence analysis. Based on the phylogenetic analysis of the whole genome, these strains formed a new independent branch in sublineage 8.7, which showed a close relationship with HP-PRRSV and intermediate PRRSV according to nucleotide and amino acid homology but displayed a completely different deletion pattern in NSP2. Recombinant analysis showed that these strains presented similar recombination patterns, all of which involved recombination with QYYZ in the ORF3 region. Furthermore, we found that the new-branch PRRSV retained highly consistent nucleotides at positions 117–120 (AGTA) of a quite conserved motif in the 3’-UTR; showed similar deletion patterns in the 5’-UTR, 3’-UTR and NSP2; retained characteristics consistent with intermediate PRRSV and exhibited a gradual evolution trend. The above results showed that the new-branch PRRSV strains may have the same origin and be similar to HP-PPRSV also evolved from intermediate PRRSV, but are distinct strains that evolved simultaneously with HP-PRRSV. They persist in some parts of China through rapid evolution, recombine with other strains and have the potential to become epidemic strains. The monitoring and biological characteristics of these strains should be further studied.

## Introduction

Porcine reproductive and respiratory syndrome (PRRS) is one of the most economically important infectious diseases of swine globally (Gao et al., 2017). The etiologic agent of PRRS has been identified as porcine reproductive and respiratory syndrome virus (PRRSV), which is currently considered to consist of two different species in the *Arteriviridae* family: *Betaarterivirus suid 1* (PRRSV-1) and *Betaarterivirus suid 2* (PRRSV-2) (Brinton et al., 2021). The disease causes reproductive failure in sows, mainly premature parturition, late-term abortions and farrowing of stillborn and nonviable piglets. Respiratory dysfunction can develop predominantly in younger pigs (Albina, 1997). Currently, the most widely prevalent strain is PRRSV-2 in North America and Asia, which is classified into nine lineages (1–9) based on phylogenetic analyses of ORF5 gene sequences collected worldwide (Shi et al., 2010), among which five main sublineages (1.5 and 1.8, 3.5, 5.1, 8.7) occur in China (Guo et al., 2018; Zhang et al., 2018; Xu et al., 2020; Yu et al., 2020).

Sublineage 8.7 was the earliest strain found in mainland China (Liu et al., 2017; Guo et al., 2018) and mainly includes three subgroups: Classical PRRSV [representing strain CH-1a (Guo et al., 1996)], intermediate PRRSV [representing strains HB-1(sh)/2002 and HB-2(sh)/2002 (Gao et al., 2004)] and highly pathogenic PRRSV [HP-PRRSV; representing strains JXA1 (Tian et al., 2007) and HuN4 (Tong et al., 2007)]. Research by An showed that HP-PRRSV appeared in China in 2006 originated from the Classical CH-1a-like PRRSV. Its evolution was based on the gradual accumulation of mutations in intermediate PRRSV strains (Zhou et al., 2009a; An et al., 2010). The pathogenicity of the viruses shows a gradually increasing trend from Classical PRRSV to intermediate PRRSV to HP-PRRSV (Zhou and Yang, 2010). Classical PRRSV and intermediate PRRSV show moderate pathogenicity (Yang et al., 2008; Li et al., 2009; Ma et al., 2009). HP-PRRSV has the strongest pathogenicity and has been one of the main circulating strains in Chinese pig herds since 2006, causing serious economic losses to the pig industry in China and Southeast Asian countries (Tian et al., 2007; Tong et al., 2007). Sublineage 1.5 and 1.8 PRRSVs have gradually replaced sublineage 8.7 PRRSV as major epidemic strains in China (Xu et al., 2022), but sublineage 8.7 strains have not disappeared, and their genetic evolution has become increasingly complex (Jiang et al., 2020). We have been paying attention to whether this branch will evolve into another new branch similar to HP-PRRSV.

In this study, a new PRRSV strain type with novel characteristics was first found in three different regions of Shandong Province during the continuous monitoring of PRRSV. These strains belonged to a new independent branch in sublineage 8.7 and showed a novel deletion pattern in the NSP2 region. Further analysis of their whole-genome characteristics alongside all strains on this branch revealed that the new branch strains presented similar characteristics to intermediate PRRSV. Furthermore, multiple unique deletions and point mutations were first found in the PRRSV genome for the first time herein, which providing an important data reference for the epidemiology of PRRSV in China.

## Materials and methods

### Sample collection

In 2020–2022, 31 clinical samples of pigs suspected of PRRSV infection, including lung, lymph node and serum samples, were collected from farms in three different regions (Qingdao, Weihai and Yantai) of Shandong Province in northern China. The morbidity caused by suspected PRRSV infection on these pig farms was approximately 10–20%, and most of the affected pigs had mixed bacterial infections. These samples were transported at low temperature and stored at −20°C. Sample names, collection times, types, isolation regions and accession numbers are summarized in Table 1.

**Table 1:** Information of new-branch PRRSV.

Isolates	Accession no.	Time	Farm	Sample types	Region	Gene region	Source
SDWH1	OQ506492	2020.09	Farm 1	Serum	Shandong Weihai	ORF5	In this study
SDWH2	OQ506493	2020.09		Serum		ORF5	
SDWH3	OQ506494 + OQ506510	2020.09		Lung/Lymph nodes		ORF5 + NSP2	
SDWH4	OQ506495	2020.09		Serum		ORF5	
SDWH5	OQ506496	2020.12		Serum		ORF5	
SDWH6	OQ506497	2020.12		Serum		ORF5	
SDWH7	OQ506498	2020.12		Serum		ORF5	
SDWH8	OQ506499 + OQ506511	2020.12		Lung/Lymph nodes		ORF5 + NSP2	
SDWH9	OQ506500	2020.12		Serum		ORF5	
SDWH32	Negative	2021.03		Lung/Lymph nodes		/	
SDWH33	Negative	2021.03		Serum		/	
SDWH55	Negative	2021.06		Serum		/	
SDWH56	Negative	2021.06		Serum		/	
SDWH81	Negative	2021.06		Lung/Lymph nodes		/	
SDWH86	OQ506501 + OQ506512	2021.06		Lung/Lymph nodes		ORF5 + NSP2	
SDWH86	OQ506516	2021.06		Lung/Lymph nodes		Whole genome	
SDWH87	OQ506502	2021.06		Serum		ORF5	
SDWH88	OQ506503	2021.06		Serum		ORF5	
SDWH89	OQ506504	2021.06		Serum		ORF5	
SDQD1	Negative	2020.12	Farm 2	Lung/Lymph nodes	Shandong Qingdao	/	
SDQD2	Negative	2020.12		Serum		/	
SDQD29	Negative	2021.03		Serum		/	
SDQD66	Negative	2021.06		Lung/Lymph nodes		/	
SDQD67	Negative	2021.06		Serum		/	
SDQD93	OQ506506	2021.08		Serum		ORF5	
SDQD94	OQ506507 + OQ506513	2021.08		Lung/Lymph nodes		ORF5 + NSP2	
SDQD95	OQ506508 + OQ506514	2021.08		Lung/Lymph nodes		ORF5 + NSP2	
SDQD95	OQ538073	2021.08		Lung/Lymph nodes		Whole genome	
SDQD96	OQ506509	2021.08		Serum		ORF5	
SDYT67	Negative	2022.06	Farm 3	Serum	Shandong Yantai	/	
SDYT68	Negative	2022.06		Serum		/	
SDYT69	Negative	2022.06		Lung/Lymph nodes		/	
SDYT91	OQ506505+OQ506515	2022.06		Lung/Lymph nodes		ORF5 + NSP2	
SDYT91	OQ538074	2022.06		Lung/Lymph nodes		Whole genome	
ZJXS1412	MF418142.1	2014.12	**–**	**–**	Zhejiang	Whole genome	GenBank
ZJXS1501	MF418145.1 + MF418166.1	2015.01	**–**	**–**	Zhejiang	ORF5 + NSP2	
ZJSX1503	MF418148.1 + MF418169.1	2015.03	**–**	**–**	Zhejiang	ORF5 + NSP2	
JS18-3	MN606304.1	2018.03	**–**	**–**	Jiangsu	Whole genome	
HB2104	MZ712110.1	2021.04	**–**	**–**	Shanghai	Whole genome	
SXS110404	JQ798268.1	2011.10	**–**	**–**	Zhejiang	ORF5	
ZJhz16-2	KF678434.1	2016.02	**–**	**–**	Zhejiang	ORF5	
ZJ/HZdt/2013	KM377861.1	2013.01	**–**	**–**	Guangdong	ORF5	
JN1504	KT961402.1	2015.04	**–**	**–**	Jiangsu	ORF5	
JSYZ1803-5	MK689113.1	2018.03	**–**	**–**	Jiangsu	ORF5	
214_HNZMD-4	MK943948.1	2019.05	**–**	**–**	Hubei	ORF5	

–, lack of related data.

### RNA extraction and RT–PCR

Tissue samples were ground with 1 ml PBS containing 2% penicillin streptomycin. Total RNA extraction from lapping fluid, cDNA preparation, RT-PCR and viral genome sequencing were performed as described previously (Xu et al., 2022). The primers used to detect PRRSV have been previously reported (Zhang et al., 2019). Seven pairs of overlapping primers were designed based on sublineage 8.7 and used to amplify the complete genome (Table 2).

**Table 2:** Primers for complete genome amplification.

Name	Primer sequence (5’-3’)	Position in the genome	Product size (bp)
HP-PRRSV-A	ATGACGTATAGGTGTTGGCTCTATG GAGCGGCTGGGATGGTACTGCTAGG	1–1,894	1894
HP-PRRSV-B	GTGAGCATTGGACTGTCTCTGTGAT ACATCCGGGGATCTTTGGCAGGTTG	1,700–3,620	1921
HP-PRRSV-C	TTTGTGATGTTACCTCGCACGCCTG ACCAATAACACCACGGCCAAGATTC	3,430–5,300	1871
HP-PRRSV-D	ATCGGAGGCATGGCTCATAGGTTGA GTGCTACCAACCTTTAGGTCGAAGA	5,106–7,010	1905
HP-PRRSV-E	CTGCGTCCAACATGAGGAATGCAGC CCGAGGGCGATGGGCGAGTTGAACG	6,780–8,700	1921
HP-PRRSV-F	AGGCTGTGCGAGAAAACTGGCAAAC TTTGTCCCTGTAATCTGGTTGAATG	8,550–10,410	1861
HP-PRRSV-G	GGCTTCTCAGTAAAACAACTCTCAC CAAACAAAATGGCCAAAAATATGAT	10,230–12,100	1871
HP-PRRSV-H	GAGGACTGGGAGGATTACAATGATG GTGCCAGCCAATCTGTGCCATTCAG	11,850–13,840	1991
HP-PRRSV-J	TGCTTCATTTCATGACACCTGAGAC TTTTTTAATTACGGCCGCATGGTTC	13,610–15,323	1714

### Sequence alignment and phylogenetic analysis

A total of 2,127 ORF5 sequences, 26 NSP2 sequences and 26 complete genomic sequences were obtained from GenBank as reference strain sequences. The nucleotide (nt) and amino acid (aa) similarity comparison of each gene fragment and the deduced amino acid sequences of the full-length genomic sequence alignment with reference strain sequences were assessed using the ClustalW method in Lasergene software (DNASTAR Inc., Madison, WI, United States) (Xu et al., 2022). Multiple sequence alignments of ORF5, NSP2, ORF3 and the whole genome were conducted using ClustalW in MEGA version 7.0 (Song et al., 2020), and phylogenetic trees were also constructed using the maximum likelihood method with 1,000 replicates and the Kimura 2-parameter substitution model (Kumar et al., 2016).

### Recombination analysis

To test for recombination, SimPlot (version 3.5.1) was applied with a 200-bp window, sliding along the genome alignments with a 20-bp step size. We also further checked for potential recombination events by using seven different algorithms (RDP, GENECONV, Boot Scan, Max Chi, chimera, Si Scan, and 4Seq) in RDP4 software (Li et al., 2022).

## Results and discussion

### Clinical sample detection

Thirty-one clinical samples from animals with suspected PRRSV infection were collected from farms in three different regions of Shandong Province in China between 2020 and 2022. Among these samples, 18 (58.1%) samples were positive for PRRSV by RT–PCR. Eighteen ORF5 sequences, 6 NSP2 gene sequences and 3 complete genomic sequences of these samples were amplified and sequenced for further analysis with reference strain sequences. Strain names, areas and accession numbers are summarized in Table 1.

### Phylogenetic analysis of ORF5 gene

**Figure 1:**
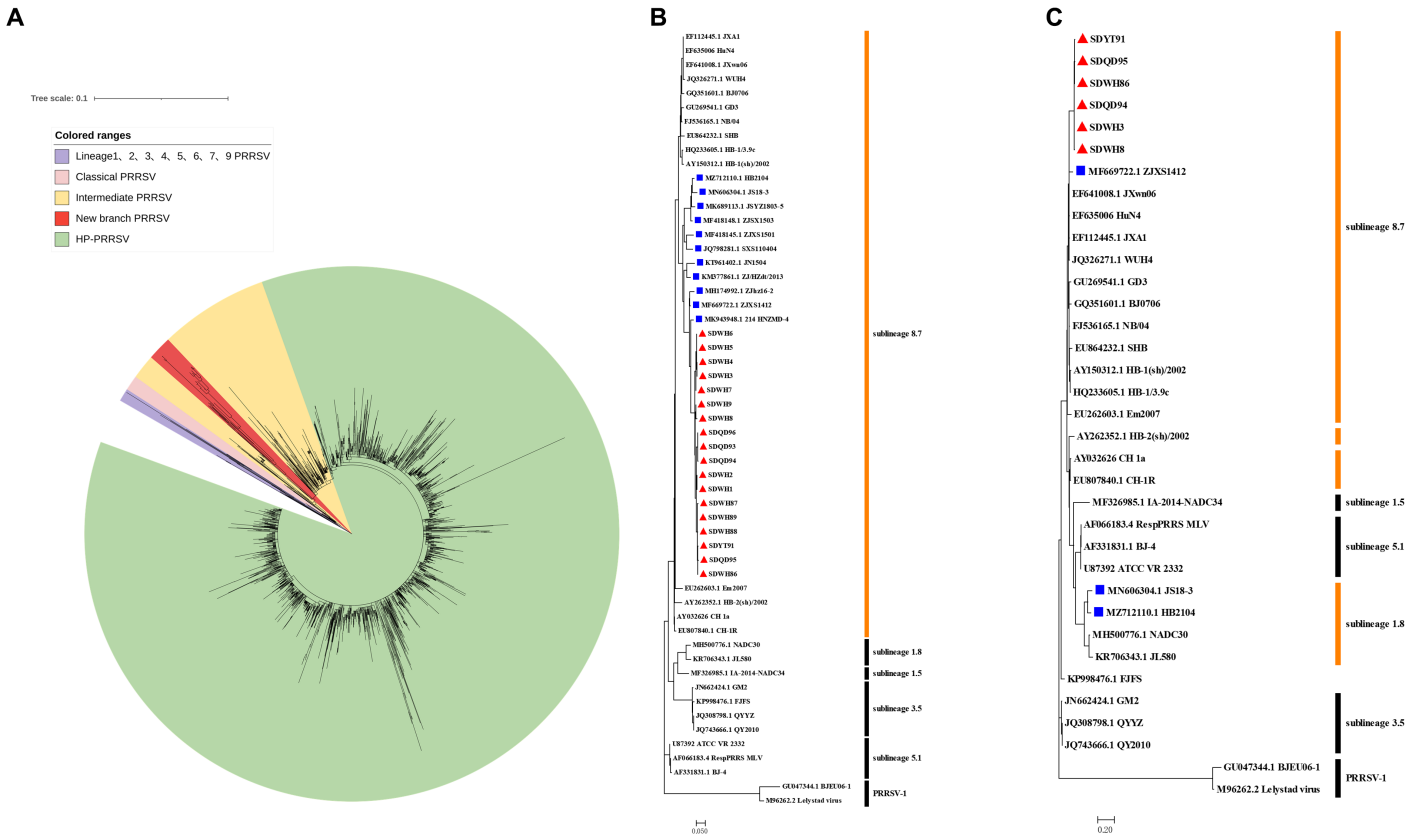
Phylogenetic analysis of GP5 and NSP2 genes of new-branch PRRSV. **(A)** Phylogenetic tree constructed based on the GenBank dataset (*n* = 2,127) of the sublineage 8.7 PRRSV ORF5 gene from China; **(B)** Phylogenetic tree constructed based on the ORF5 gene of new-branch PRRSV isolates and reference strains from different sublineages; **(C)** Phylogenetic tree constructed based on the NSP2 gene of new-branch PRRSV isolates and reference strains from different sublineages. The new-branch PRRSV strains obtained in this study are labeled with red triangles (▲). The strains obtained from GenBank belonging to the new branch are marked with blue squares (■).

In the construction phylogenetic trees of PRRSV, the ORF5 sequence is often used as the ideal target (Shi et al., 2010). Sequencing of GP5 revealed that these strains belonged to sublineage 8.7 PRRSV (data not shown). To understand the evolutionary relationships of these newly obtained PRRSV strains with representative strains in China, phylogenetic trees based on the sublineage 8.7 PRRSV ORF5 gene sequence set (2127) collected from GenBank was constructed using the maximum likelihood method in MEGA version 7.0. The results demonstrated that all 18 new PRRSV strains in this study formed a new independent branch within sublineage 8.7 and belonged to transitional PRRSV strains between intermediate PRRSV and HP-PRRSV (Figure 1A). To visualize the results, all strains of the new branch are displayed in the ORF5 phylogenetic tree in Figure 1B. It is worth noting that some sequences from GenBank were also assigned to this new branch, and only the ZJXS1412, HB2104 and JS18-3 strains had complete genome information. To explore the origin and evolution of the new-branch strains, we also included these strains from GenBank in our subsequent analysis. Information of these strains is shown in Table 1.

### Phylogenetic and sequence alignment analyses of NSP2 gene

**Figure 2:**
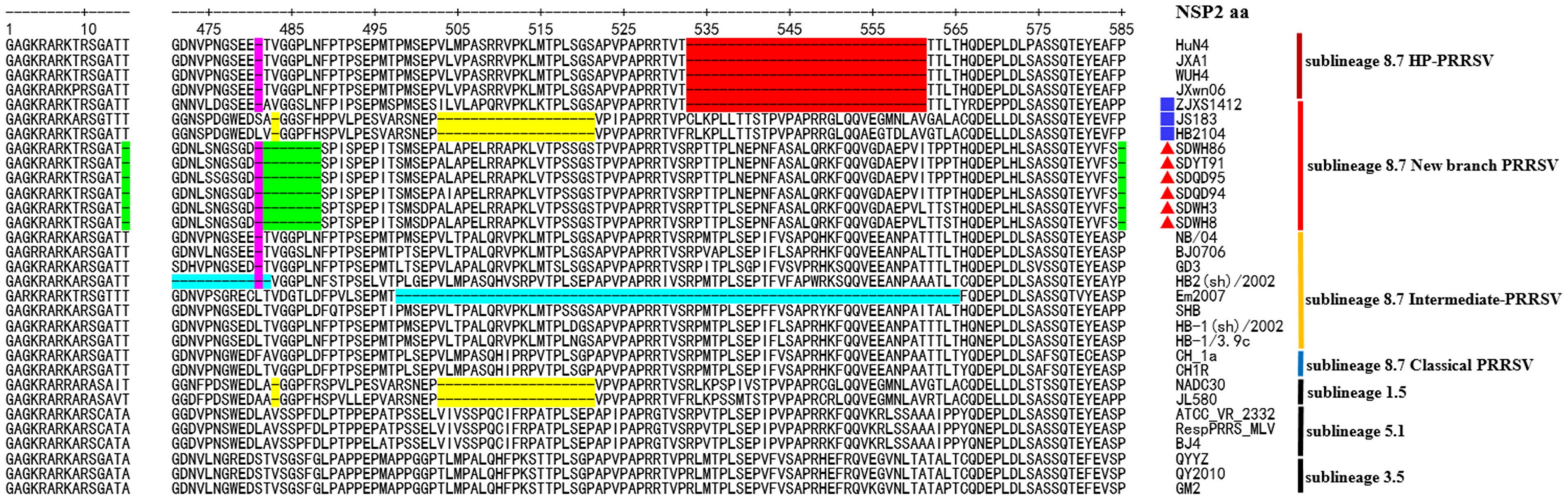
The deduced NSP2 amino acid sequences of the new-branch strains compared with the reference PRRSV strains. The position of the deduced Nsp2 amino acid sequence relative to the VR-2332 strain. Purple indicates the common aa deletion of intermediate PRRSV, new-branch PRRSV and HP-PRRSV. Red indicates the deletion pattern of HP-PRRSV; green indicates the novel characteristic discontinuous deletion pattern of new-branch PRRSV; yellow indicates the deletion pattern of NADC30 PRRSV (not fully displayed); cyan indicates other deletion patterns of intermediate PRRSV strains. The new-branch PRRSV strains obtained in this study are labeled with red triangles (▲). The strains obtained from GenBank belonging to the new branch are marked with blue squares (■).

In recent years, an increasing number of studies have reported that the recombination events continue to increase in PRRSV in China (Yu et al., 2020; Chen et al., 2021). Therefore, the PRRSV classification criteria only based on the ORF5 gene alone may not be rigorous. The Nsp2 gene is one of the most variable regions in the entire PRRSV genome (Nelsen et al., 1999), which can tolerate some amino acid deletion, insertion or mutation and it is commonly usually used as a basis for classifying different strains. According to our phylogenetic analysis based on the NSP2 gene, ZJXS1412 from GenBank and 6 new PRRSV strains (SDWH86, SDQD95, SDYT91, SDQD94, SDHW3, and SDHW8) were classified into sublineage 8.7 and formed a relatively independent branch parallel to the HP-PRRSV branch, while HB2104 and JS18-3 from GenBank belonged to sublineage 1.8 (Figure 1C). However, amino acid alignment based on the Nsp2 gene results showed that the new-branch strains displayed a completely different deletion pattern in the Nsp2 region from other sublineage 8.7 strains, retained characteristics consistent with intermediate PRRSV, and presented a gradual evolutionary trend. Most of the new-branch PRRSV, intermediate PRRSV and HP-PRRSV strains showed a deletion at the 481th aa of NSP2 relative to the reference strain VR-2332 (Figure 2). JS18-3 and HB2104 showed the same deletion pattern with the NADC30 strain (111 aa+1 aa+19 aa) due to recombination with the NADC30 strain (Han et al., 2020). ZJXS1412 maintained the same deletions at positions 481 and 533–561 (1 aa+29 aa) as HP-PRRSV. SDWH86, SDQD95 and SDYT91 showed further deletions at positions 15, 482–488 and 583 on the basis of the 481st aa deletion. To the best of our knowledge, this is a novel deletion pattern (1 aa+8 aa+1 aa) that has not been previously reported in sublineage 8.7 PRRSV in China. Although studies have shown that the 30 aa deletion in Nsp2 of the HP-PRRSV virus observed in China is not related to its virulence (Zhou et al., 2009b), some new discoveries about the function of NSP2 have been reported recently (Kappes et al., 2013; Morgan et al., 2013; Song et al., 2019; Chen et al., 2022). Therefore, the effect of the new deletion pattern of NSP2 found in this study needs to be further explored based on an infectious clone platform. At present, many major epidemic PRRSV strains in China have formed relatively stable and unique characteristics in the NSP2 region, such as a discontinuous 30 aa (1 aa+29 aa) deletion in Nsp2 that is used as the gene marker distinguishing HP-PRRSV from other strains (Tian et al., 2007). Similarly, NADC30-like PRRSV and NADC34-like PRRSV have discontinuous 131 aa deletion (111 aa+1 aa+19 aa) and 100 aa deletion in Nsp2, respectively (Xu et al., 2020). Therefore, excluding recombinant strains such as JS18-3 and HB2104, this new-branch PRRSV with a 1 + 8 + 1 aa stable deletion in NSP2 may represent a necessary condition for becoming a mainstream strain. This further illustrates that the Nsp2 region of sublineage 8.7 PRRSV displays more complex deletion patterns in China.

### Homology analysis of full-genome

In view of the unique characteristics of GP5 and NSP2, we further analyzed the full-length genomic characteristics of these PRRSV strains belonging to the new branch. The full-length genome sequences of 3 new PRRSV strains (SDWH86, SDQD95, and SDYT91) consisted of 15,378 nt, a 188-nt 5′-untranslated region (5’-UTR) and a 148-nt 3′-UTR. The full-length genomic nucleotide sequence homology percentages of these sequences with representative PRRSV strains HuN4, CH-1a, HB-1(sh)/2002, VR-2332, NADC30 and QYYZ were 94.0–94.2%, 91.6–91.7%, 92.8–92.9%, 87.5–87.7%, 83.6–83.8% and 85.1–86.1%, respectively. The 5′-UTR, ORF1a, ORF1b, ORF5, and ORF7 shared the highest homology with the HP-PRRSV HuN4 strain; ORF2a, ORF4, ORF5a and ORF6 shared the highest homology with the intermediate PRRSV HB-1(sh)/2002 strain; ORF2b shared the highest homology with the Classical PRRSV CH-1a strain; ORF3 shared the highest homology with the QYYZ strain; and the 3′-UTR shared the highest homology with the NADC30 strain (Table 3). Considering the high homology resulting from the evolutionary relationships among Classical PRRSV, intermediate PRRSV and HP-PRRSV strains (An et al., 2010), we speculated that these strains may show recombination in the ORF3 region.

**Table 3:** Nucleotide and amino acid sequence similarity between new PRRSV and reference strains.

Nucleotide/amino acid	CH-1a	HB-1(sh)/2002	HuN4	ATCC-VR2332	NADC30	QYYZ
5′UTR	95.2–95.7	96.3–96.8	98.9–99.5	92.6–93.1	92.6–93.1	95.2–95.7
Nsp1α	94.1–94.4/95.6	95.0–95.4/96.1	96.9–97.2/97.8	90.9–91.1/95.6	88.5–88.7/95.6	91.3–91.7/96.1
Nsp1β	90.3–90.6/86.1–87.1	93.9–94.2/94.1–95.0	96.5–96.9/95.5–96.5	85.0–85.3/82.7–83.7	80.2–80.7/75.7–76.2	80.484.3/81.7–82.7
Nsp2	88.6–88.8/84.0–84.3	91.0–91.3/88.0–88.4	93.0–93.2/90.8–91.0	82.6–82.7/77.3–77.6	75.8–75.9/71.0–71.1	81.2–81.3/76.2–76.4
Nsp3	92.5–92.6/97.8–98.3	94.1–94.2/97.0–97.4	96.8–97.0/98.7–99.1	88.8–89.0/94.8–95.2	82.9–83.0/90.9–91.3	80.3–80.4/87.4–87.8
Nsp4	93.5–93.8/96.1–96.6	95.8–96.1/98.0–98.5	97.1–97.4/99.0–99.5	90.4–90.7/93.6–94.1	84.8–85.0/92.2–92.6	84.3–84.5/91.7–92.2
Nsp5	93.7–93.9/94.7	96.1–96.3/98.8	97.8–98.0/98.8	88.8/91.2	90.4–90.6/94.7	81.4–81.6/89.4
Nsp6	95.8/100.0	97.9/100.0	97.9/100.0	93.8/93.8	91.7/93.8	95.8/100.0
Nsp7α	95.1–95.5/96.0–96.6	95.7–96.2/97.3–98.0	97.3–97.8/98.0–98.7	88.8–89.3/94.0–94.6	82.6–83.0/90.6–91.3	90.6–91.1/93.3–94.0
Nsp7β	90.3/91.8	93.0/91.8	96.1/95.5	85.5/80.0	77.6/75.5	91.8/91.8
Nsp8	96.3–97.0/95.6–97.8	94.8–95.6/93.3–95.6	95.6–96.3/95.6–97.8	94.1–94.8/95.6–97.8	86.7–87.4/91.1–93.3	94.1–94.8/95.6–97.8
Nsp9	94.1–94.2/97.5–97.7	94.9–95.0/98.1–98.3	95.6–95.7/98.3–98.4	90.0–90.1/96.7–96.9	86.8–86.9/96.7–96.9	90.3–90.4/96.7–96.9
Nsp10	91.8–91.9/96.8–97.3	92.4–92.6/97.5–98.0	94.2–94.3/98.2–98.6	88.0–88.1/95.5–95.9	84.8–85.1/94/8–95.2	87.2–87.5/96.1–96.6
Nsp11	91.2–91.5/97.8–98.2	91.6–91.9/96.9–97.3	92.4–92.7/97.3–97.8	87.6–88.0/94.2–94.6	88.2–88.6/95.5–96.0	86.7–87.0/93.7–94.2
Nsp12	94.0–94.2/95.5–96.1	94.2–94.4/95.5–96.1	95.7–95.9/98.1–98.7	87.0–87.3/93.5–94.2	89.2–89.4/94.2–94.8	86.6–86.8/94.8–95.5
ORF2a	90.0–90.4/89.1–89.9	90.3–90.5//89.1–89.9	89.0–89.4/88.3–89.1	89.1–89.5/89.1–89.9	85.9–86.3/86.4–87.2	86.8–87.2/86.4–87.2
ORF2b	95.0–95.9/89.2–91.9	94.1–94.6/90.5–91.9	93.7–94.6/89.2–91.9	93.2–94.1/87.8–90.5	91.0/86.5–89.2	90.5–91.4/85.1–87.8
ORF3	86.3–86.4/83.1–83.5	86.8–86.9/83.5–83.0	85.8–85.9/82.7–83.1	86.9–87.1/83.1–83.5	81.6–81.7/81.2–81.6	87.5–87.6/85.5–85.9
ORF4	91.4/89.4	91.4/89.9	91.1/89.9	90.1/89.9	87.3/87.7	88.8/87.7
ORF5	89.6–90.5/88.1–90.0	90.4–91.2/88.1–90.5	91.4–92.2/89.1–91.5	85.7–86.1/83.1–85.1	84.7–85.9/85.6–88.1	82.1–82.6/78.6–80.6
ORF5a	89.7–90.4/84.6	89.1–89.7/88.5–94.0	89.1–89.7/84.6–86.5	87.287.8−/76.9–78.8	86.5–88.5/76.9–78.8	83.3–84.0/71.2–73.1
M	93.0–93.3/92.6–93.7	94.9–95.4/94.9–96.0	95.0–95.4/94.3–95.4	93.3–93.9/94.3–96.0	87.4–88.0/91.4–93.1	88.8–89.1/92.0–93.1
N	92.7/95.2	93.0/95.2	93.3/95.2	92.5/95.2	90.1/91.1	89.8/92.7
3’UTR	83.9–85.8	81.9–83.8	85.9–87.8	85.9–87.8	87.2–89.2	83.9–85.8
Whole genome	91.6–91.7	92.8–92.9	94.0–94.2	87.5–87.7	83.6–83.8	85.1–86.1

### Recombination and phylogenetic analysis of full-genome

**Figure 3:**
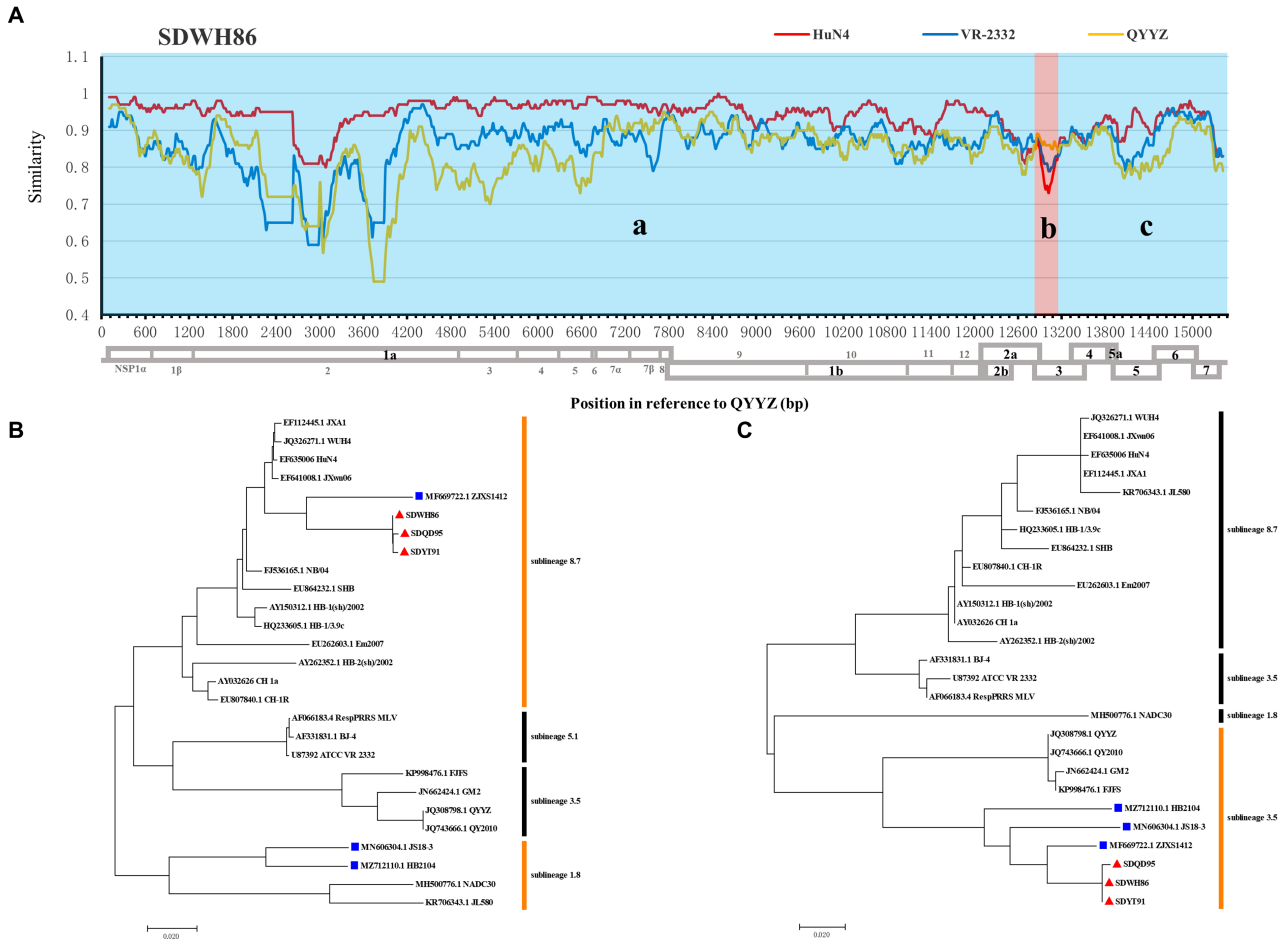
Genome recombination analysis of the SDWH86 isolate. **(A)** Similarity plots generated with the representative strains HuN4, VR-2332 and QYYZ by Simplot v3.5.1; **(B)** Phylogenetic trees based on recombinant fragment a + c within SDWH86 and reference PRRSV strains; **(C)** Phylogenetic trees based on recombinant fragment b within SDWH86 and reference PRRSV strains. The blue region (a + c fragment) indicates that the major parent was HP-PRRSV, and the red region (b fragment) indicates that the minor parent was QYYZ. The new-branch PRRSV strains obtained in this study are labeled with red triangles (▲). The strains obtained from GenBank belonging to the same branch are marked with blue squares (■).

**Figure 4:**
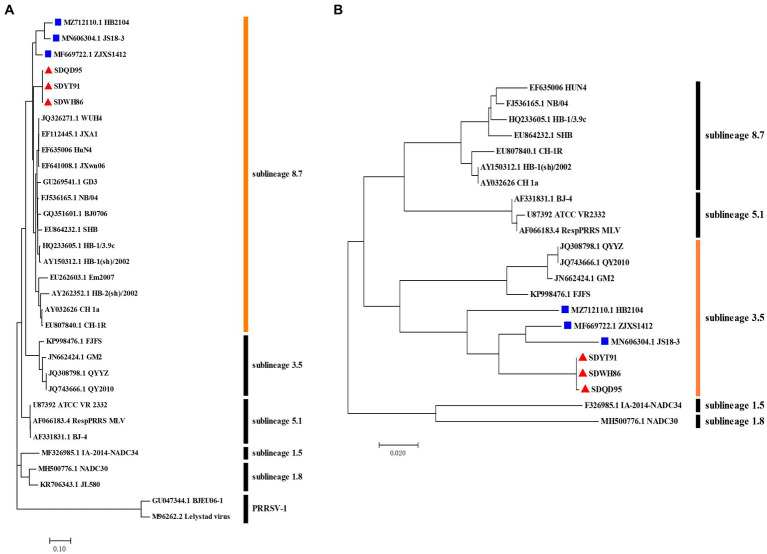
Phylogenetic analysis of the whole genome and ORF3 gene of new-branch PRRSV. **(A)** Phylogenetic tree constructed based on the whole genome of new-branch PRRSV isolates and reference strains from different sublineages; **(B)** Phylogenetic tree constructed based on the ORF3 gene of new-branch PRRSV isolates and reference strains from different sublineages. The new branch PRRSV strains obtained in this study are labeled with red triangles (▲). The strains obtained from GenBank belonging to the new branch are marked with blue squares (■).

Indeed, recombination analysis based on RDP4 and SimPlot software verified our conjecture. The results of RDP4 analysis showed that the new-branch strains (SDWH86, SDQD95, and SDYT91) were recombinant viruses with two different sources, among which HP-PRRSV HuN4 was the major parental strain, while QYYZ was the minor parental strain (Table 4). It is worth noting that the RDP4 software analysis showed that HP-PRRSV was the major parental strain, but the homology comparison results showed that these new PRRSV strains presented some degree of homology with the intermediate strains, and their NSP2 region did not have the same characteristics as that of HP-PRRSV. Therefore, their major parents may have evolved from intermediate strains and been homologous to HP-PRRSV, but we have not yet found them. And SimPlot results were similar to those of RDP4. Taking the SDWH86 strain as an example, two recombination breakpoints identified based on RDP4 were used to divide the whole genome into three regions: 1–12,885 (a), 12,885–13,056 (b), and 13,056–15,378 (c) (Figure 3A). Phylogenetic analysis based on breakpoints provided consistent results. The a + c segment belonged to sublineage 8.7 (Figure 3B), and the b segment belonged to sublineage 3.5 (Figure 3C). Therefore, SDWH86, SDQD95 and SDYT91 are recombinant viruses positioned between sublineage 8.7 and 3.5. However, the ZJXS1412, HB2104 and JS18-3 strains on the new branch have more complex recombination patterns (Supplementary Table S1). ZJXS1412 is a sublineage 8.7, 3.5 and 5.1 recombinant; JS18-3 is a sublineage 8.7, 3.5 and 1.8 recombinant; HB2104 is a sublineage 8.7, 3.5, 1.8 and 5.1 recombinant (Supplementary Figure S1). Further phylogenetic analysis based on full-length genomes showed that the SDWH86, SDQD95, SDYT91, ZJXS1412, HB2104, and JS18-3 isolates formed independent branches in sublineage 8.7 (Figure 4A). Additionally, phylogenetic analysis based on the GP3 gene showed that they all belonged to sublineage 3.5, which was a common characteristic of the new-branch strains (Figure 4B). Our previous study described the possible mechanism of complex recombination pattern formation (Xiang et al., 2022). Therefore, we speculated that the complex recombination pattern of the new-branch PRRSV ZJXS1412, HB2104, and JS18-3 strains evolved from the parental strain with sublineage 8.7 and 3.5 recombination, which provided skeletons or fragments for recombination and continuously produced more complex recombination patterns.

**Table 4:** Information of recombination events of three new PRRSV detected by RPD4 software.

Strains	Breakpoints		Parental sequence		Detection methods (*p*-value)						
	Beginning	Ending	Major	Minor	RDP	GENECONV	BootScan	MaxChi	Chimaera	SiScan	3Seq
SDWH86	12,885	13,056	HuN4	QYYZ	1.642 × 10^−6^	1.686 × 10^−2^	–	9.416 × 10^−3^	1.462 × 10^−3^	–	1.862 × 10^−2^
SDQD95	12,935	13,063	HuN4	QYYZ	–	1.459 × 10^−2^	–	1.317 × 10^−2^	7.518 × 10^−3^	–	1.366 × 10^−2^
SDYT91	12,885	13,055	HuN4	QYYZ	1.950 × 10^−10^	3.542 × 10^−4^	–	1.249 × 10^−2^	5.332 × 10^−3^	2.765 × 10^−2^	9.914 × 10^−5^

–, not significant.

### Sequence alignment analysis of 5’-UTR and 3’-UTR

**Figure 5:**
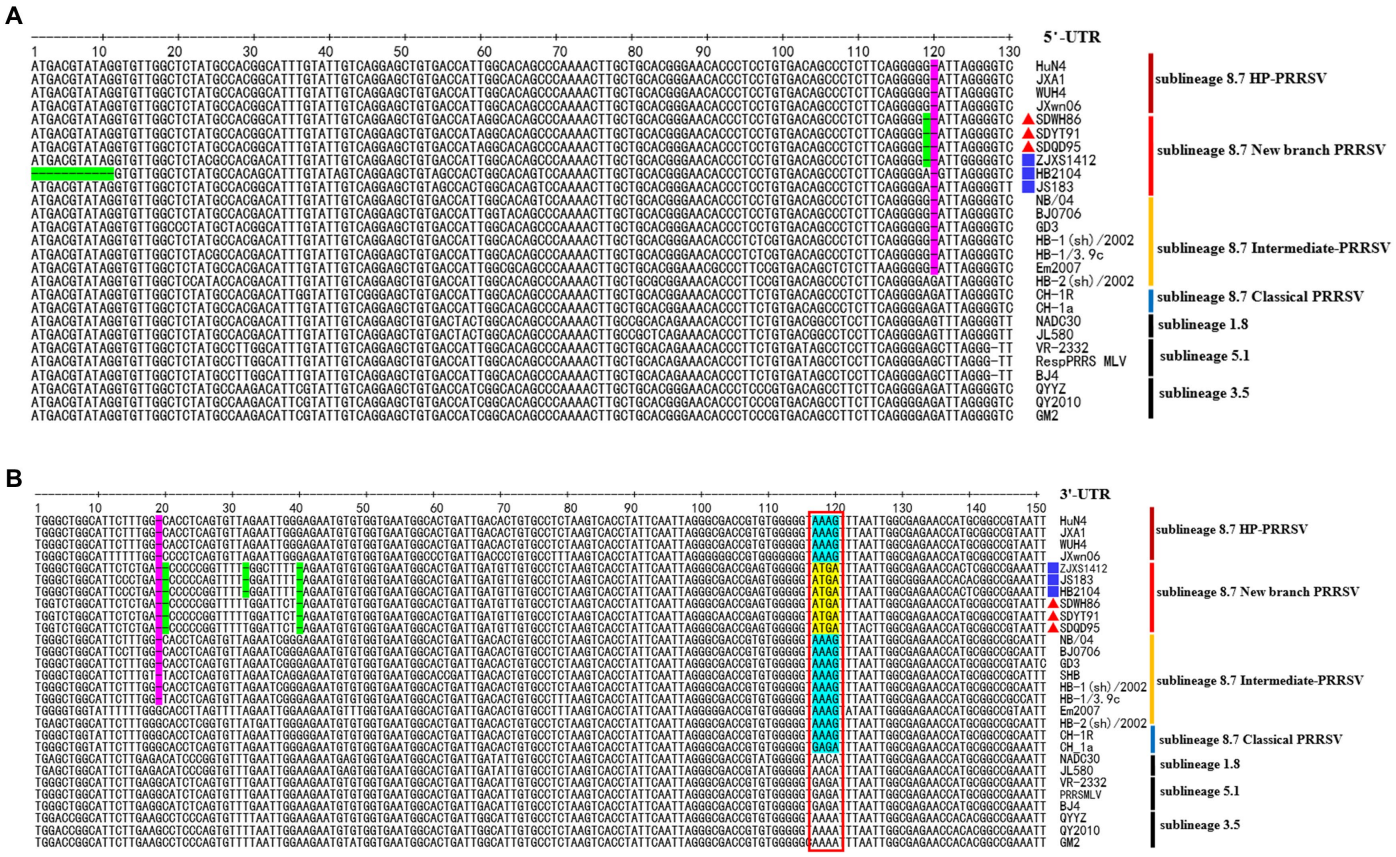
The deduced 5’-UTR and 3’-UTR nucleotide sequences of the new-branch strains compared with the reference PRRSV strains. **(A)** The positions of the deduced 5’-UTR nucleotide sequences relative to the VR-2332 strain. **(B)** The positions of the deduced 3’-UTR nucleotide sequences relative to the VR-2332 strain. Purple indicates the common nt deletions of intermediate PRRSV, new-branch PRRSV and HP-PRRSV, while green indicates new deletions on this basis; a red box indicates a highly conserved motif at positions 117–120 in the 3’-UTR, yellow represents the motif of the new-branch PRRSV, and cyan represents the motif of the Classical strain, intermediate strain and HP-PRRRSV. The new-branch PRRSV strains obtained in this study are labeled with red triangles (▲). The strains obtained from GenBank belonging to the new branch are marked with blue squares (■).

Sequence alignment analysis showed that the new-branch PRRSV strains had similar characteristics in their 5′-UTR and 3′-UTR. They not only retained the same deletion pattern characteristics as intermediate PRRSV strains but also showed a trend of gradual evolution (Figure 5). In the 5′-UTR, new-branch PRRSV, intermediate PRRSV and HP-PRRSV all presented a deletion at the 120th nucleotide (Figure 5A). JS18-3 and HB2104 maintained the same deletion at the 120th nucleotide position as most intermediate PRRSV and HP-PRRSV strains, while SDWH86, SDQD95, SDYT91 and ZJXS1412 showed a continuous 2-nucleotide deletion at positions 119 and 120. The continuous deletion of two nucleotides from the 119th to 120th positions in the 5′-UTR has been previously reported (Zhou et al., 2011; Shi et al., 2013). Since the PRRSV 5′-UTR plays a crucial role in replication, mRNA transcription and protein translation (Gao et al., 2012), the effect of additional deletions in the 5′-UTR needs further study. In the 3′-UTR, new-branch PRRSV, intermediate PRRSV and HP-PRRSV all had a deletion at the 19th nucleotide (Figure 5B). Even more interestingly, SDWH86, SDQD95 and SDYT91 showed further deletion of the 20th and 40th nucleotides. This is the first report of a novel deletion pattern in the 3′-UTR of sublineage 8.7 PRRSV in China. JS18-3, HB2104 and ZJXS1412 showed further deletion of the 32nd nucleotide on the basis of the 19-20th and 40th nucleotide deletions. In addition, the new-branch PRRSV strains presented a completely different motif (ATGA) from the 117-120th nucleotides of the 3′-UTR compared to the sublineage 8.7 strains (Figure 5B). The most recent research indicated that the motif of the 117-120th nucleotides in the 3′-UTR is quite conserved within each sublineage of PRRSV; for example, in most sublineage 8.7 strains, this motif is AAAG (in HP-PRRSVs) or GAGA (in Classical PRRSVs) (Xiong et al., 2022). Some mutations in this motif have been confirmed to enhance the replication ability of HP-PRRSV or L1 PRRSV (Xiong et al., 2022). Therefore, we speculated that the new-branch strains with the same motif in the 3′-UTR may have the same origin; although this motif is inconsistent with those of the sublineage 8.7 strains, it may be a result of evolution. This finding contradicts our previous understanding of sublineage 8.7 PRRSV once again.

### The origin and evolution analysis of the new-branch PRRSV

To deepen the understanding of the origin and genetic relationships of the new-branch PRRSV strains and identify common points in their genome evolution shared with sublineage 8.7 PRRSV, we further collated and analyzed all available the information of the new-branch strains (Supplementary Table S1) and compared the characteristics of the new-branch PRRSV and sublineage 8.7 PRRSV strains (Classical PRRSV, intermediate PRRSV, and HP-PRRSV) from China (Table 5). Previous studies have shown that HP-PRRSV originated from the Classical CH-1a-like PRRSV. This process is formed on the gradual accumulation of intermediate PRRSV strains (An et al., 2010). The new-branch PRRSV strains shared many common characteristics with intermediate PRRSV in the 5′-UTR, 3′-UTR and NSP2 and displayed a gradual evolution trend. The regular deletions in these regions indicated that the new-branch PRRSV strains may have evolved from intermediate PRRSV, and these deletions might have evolved in a step-by-step manner. Furthermore, the results of nucleotide and amino acid homology analyses showed that the new-branch PRRSV strains presented higher homology with the HP-PPRSV strains. However, the NSP2 region of most new-branch strains did not have the same characteristics as that of HP-PPRSV strains except for ZJXS1412, while ORF2a, ORF4, ORF5a and ORF6 of the new-branch strains shared the highest homology with the intermediate PRRSV HB-1(sh)/2002 strain. Therefore, we speculated that the new-branch PRRSV strains may have the same origin and be similar to HP-PRRSV also evolved from intermediate PRRSV, but they constituted separate strains that evolved simultaneously with HP-PRRSV. The new-branch strains showed three different evolutionary directions: some strains, such as ZJXS1412, evolved the same 1 aa+29 aa deletion pattern as HP-PRRSV; some strains, such as SDWH86, SDQD95, and SDYT91, evolved a completely different deletion pattern (1 aa+8 aa+1 aa) from HP-PRRSV but also maintained a close relationship with HP-PRRSV; and some strains, such as HB2104 and JS18-3, displayed a complex recombination pattern. Based on the limited background information obtained from GenBank, these strains of this new-branch could be traced back to 2011. They persist in some areas of China, continue to evolve based on new mutation and recombination events and have the potential to become epidemic strains. At present, new-branch strains have appeared in six provinces, including Shandong, Zhejiang, Jiangsu, Shanghai, Guangdong and Hubei. Unfortunately, the new-branch strains found in this study could not be isolated from primary PAM cells or passaged Marc-145 cells, and their pathogenicity needs further verification. To date, only the JS18-3 strain has been systematically evaluated. It exhibits a relatively high replication ability and severe cytopathic effects *in vitro* and presents higher virulence in pigs than low-pathogenicity CH-1a-like field strains (Han et al., 2020). This result shows similarity to the characteristics of reported intermediate strains in terms of pathogenicity and cell tropism (Yang et al., 2008; Li et al., 2009; Ma et al., 2009). Therefore, the possibility of the new-branch strains further producing new strains with stronger pathogenicity based on mutation and recombination cannot be ruled out. This highlights the importance of the continuous monitoring of sublineage 8.7 PRRSV in China, especially these strains with novel characteristics.

**Table 5:** Comparison of characteristics between new-branch PRRSV and sublineage 8.7 PRRSV in China.

	Classical PRRSV	Intermediate PRRSV	New-branch PRRSV	HP-PPRSV
Whether formed a new sublineage?	Yes	Yes	Yes	Yes
Whether it is prevalent in the China?	Yes	Yes	Yes	Yes
Major NSP2 deletion mode	None	Most None or 1 (481th aa)	1 + 8 + 1 (15, 481–488,583th aa)	1 + 29 (481 + 532-561th aa)
Major 5’UTR deletion mode	None	None or 1 (120th nt)	1 + 1 (119–120th nt)	1 (120th nt)
Major 3’UTR deletion mode	None	None or 1 (19th nt)	2 + 1 (19–20, 40th nt)	1 (19th nt)
3’-UTR conserved motif	AAAG/GAGA	AAAG	ATGA	AAAG
Possible origins	Imported from USA	Originated from Classical PRRSV	Originated from Intermediate PRRSV	Originated from Classical PRRSV, Intermediate PRRSV as transition (An et al., 2010)
Is there recombination of early strains?	None	None	Yes	None
Province or region of early strains distributed	Beijing and North of China	Hebei and North of China	Zhejiang and Southeast of China	Jiangxi and South of China
Background of early strains emergence	None	None	The emergence of Lineage1 and Lineage3 strains	None
Pathogenicity	**+** (Han et al., 2020)	**++** (Yang et al., 2008)	**++** (Han et al., 2020)	**+++** (Tian et al., 2007)
Cell tropism to Marc-145 cell	**–**	−**/+** (Ma et al., 2009)	**++** (Han et al., 2020)	**+++** (Tong et al., 2007)

Plus sign (**+**) indicates the intensity of pathogenicity or cell tropism to Marc-145 cell. The minus sign (**−**) indicates no pathogenicity or cell tropism to Marc-145 cell.

## Conclusion

In summary, we identified a new type of PRRSV strain with novel deletion patterns in the 5′-UTR, 3′-UTR and NSP2 regions, which showed the same origin and formed a new independent branch in sublineage 8.7. They may be similar to HP-PRRSV also evolved from intermediate PRRSV, but represent separate strains that evolved simultaneously with HP-PRRSV. In addition, they have undergone rapid evolution and recombined with other strains and have the potential to become epidemic strains, which should receive more attention.

## Data availability statement

The datasets presented in this study can be found in online repositories. The names of the repository/repositories and accession number (s) can be found in the article/Supplementary material.

## Ethics statement

This study was approved by the Animal Ethics Committee of the School of Harbin Veterinary Research Institute of the Chinese Academy of Agricultural Sciences and was performed in accordance with animal ethics guidelines and approved protocols. The Animal Ethics Committee approval number was SYXK (Hei) 2011022.

## Author contributions

HZ, QW, and ZT: conceived and designed the experiments. WL, CLi, and ZG: performed the experiments. HX, BG, QS, JZ, LX, CLe, JP, GZ, YT, and HL: sample collection and data curation. WL, HZ, and QW: contributed to the writing of the manuscript. TA, XC, and ZT: reviewed and edited the manuscript. All authors contributed to the article and approved the submitted version.

## Funding

This study was supported by grants from the National Key Research and Development Program (grant no. 2022YFF0711004), the Natural Science Foundation of Heilongjiang Province (grant no. YQ2022C042), the National Natural Science Foundation of China (grant nos. 32002315 and 32172890), the State Key Laboratory of Veterinary Biotechnology Foundation (grant no. SKLVBF202208) and the National Center of Technology Innovation for Pigs.

## Conflict of interest

The authors declare that the research was conducted in the absence of any commercial or financial relationships that could be construed as a potential conflict of interest.

## Publisher’s note

All claims expressed in this article are solely those of the authors and do not necessarily represent those of their affiliated organizations, or those of the publisher, the editors and the reviewers. Any product that may be evaluated in this article, or claim that may be made by its manufacturer, is not guaranteed or endorsed by the publisher.
