# Supplementary material for: Genomic characteristics of a novel emerging PRRSV branch in sublineage 8.7 in China
Source: Front Microbiol. 2023 May 31;14:1186322. doi: 10.3389/fmicb.2023.1186322 (PMC10264644; doi:10.3389/fmicb.2023.1186322)
Supplement: Supplementary file 1 [file Data_Sheet_1.docx]

Supplementary Material

**Genomic characteristics of a novel emerging PRRSV branch in sublineage 8.7 in China**

Running title: A novel emerging PRRSV branch in sublineage 8.7 in China

Wansheng Li ^1†^, Chao Li ^1†^, Zhenyang Guo ^1†^, Hu Xu ^1^, Bangjun Gong ^1^, Qi Sun ^1^, Jing Zhao ^1^, Lirun Xiang ^1^, Chaoliang Leng ^2^, Jinmei Peng ^1^, Guohui Zhou ^1^, Yan-dong Tang ^1^, Huai-ran Liu^1^, Tongqing An ^1^, Xue-Hui Cai ^1^, Zhi-Jun Tian ^1^, Qian Wang ^1^*, Hongliang Zhang ^1^**

1^1^ State Key Laboratory of Veterinary Biotechnology, Harbin Veterinary Research Institute, Chinese Academy of Agricultural Sciences, Harbin 150001, China

^2^ Henan Key Laboratory of Insect Biology in Funiu Mountain, Henan Provincial Engineering Laboratory of Insects Bio-reactor, China-UK-NYNU-RRes Joint Laboratory of Insect Biology, Nanyang Normal University, Nanyang 473061, China

^†^ These authors contributed equally to this work.

* Corresponding author contact: Harbin Veterinary Research Institute, CAAS, No. 678 Haping Road, Xiangfang District, Harbin 150001, China

Tel.: +86-18345721188

E-mail address: [wangqian@caas.cn](mailto:tianzhijun@caas.cn)

**Corresponding author contact: Harbin Veterinary Research Institute, CAAS, No. 678 Haping Road, Xiangfang District, Harbin 150001, China

Tel.: +86-13624503578

E-mail address: [zhanghongliang01@caas.cn](mailto:tianzhijun@caas.cn)

# Table S1 Genomic characteristics of all strains in the same new branch

| Taxa | Isolation date | Isolation/Submission regions | Gene region | Recombination with | Recombination regions | Genomic characteristics | | | | References |
| --- | --- | --- | --- | --- | --- | --- | --- | --- | --- | --- |
|  |  |  |  |  |  | 5’-UTR deletion mode | 3’-UTR deletion mode | 3’-UTR 117–120th nucleotides | NSP2 deletion mode |  |
| HuN4 | 2007 | Hunan | Whole genome | NO | NO | 1 (nt: 120th) | 1 (nt: 19th) | AAAG | 1+29 | Reference |
| SDWH86 |  | Shandong | Whole genome | Lineage 8.7+3.5 | ORF3 | 1+1 (nt: 119-120th) | 2+1 (nt: 19-20, 40th) | ATGA | 1+8+1 | This study |
| SDQD95 |  | Shandong | Whole genome | Lineage 8.7+3.5 | ORF3 | 1+1 (nt: 119-120th) | 2+1 (nt: 19-20, 40th) | ATGA | 1+8+1 | This study |
| SDYT91 |  | Shandong | Whole genome | Lineage 8.7+3.5 | ORF3 | 1+1 (nt: 119-120th) | 2+1 (nt: 19-20, 40th) | ATGA | 1+8+1 | This study |
| SDWH3 |  | Shandong | ORF5+NSP2 | **-** | **-** | **-** | **-** | **-** | 1+8+1 | This study |
| SDWH8 |  | Shandong | ORF5+NSP2 | **-** | **-** | **-** | **-** | **-** | 1+8+1 | This study |
| SDQD94 |  | Shandong | ORF5+NSP2 | **-** | **-** | **-** | **-** | **-** | 1+8+1 | This study |
| ZJXS1412 | 2014.12 | Zhejiang | Whole genome | Lineage 8.7+3.5+5.1 | NSP7b-NSP9;ORF3 | 1+1 (nt: 119-120th) | 2+1+1 (nt: 19-20, 32, 40th) | ATGA | 1+29 | **-** |
| ZJXS1501 | 2015.01 | Zhejiang | ORF5+NSP2 | **-** | **-** | **-** | **-** | **-** | 1+29 | **-** |
| ZJSX1503 | 2015.03 | Zhejiang | ORF5+NSP2 | **-** | **-** | **-** | **-** | **-** | 1+29 | **-** |
| JS18-3 | 2018.03 | Jiangsu | Whole genome | Lineage 8.7+3.5+1.8 | Nsp2-Nsp3, ORF3 | 1 (nt: 120th) | 2+1+1 (nt: 19-20, 32, 40th) | ATGA | 111+1+19 | 31975574 |
| HB2104 | 2021.04 | Shanghai | Whole genome | Lineage 8.7+3.5+1.8+5.1 | Nsp2-Nsp6; NSP7b-NSP9;ORF2a-ORF4; | 1 (nt: 120th) | 2+1+1 (nt: 19-20, 32, 40th) | ATGA | 111+1+19 | **-** |
| SXS110404 | 2011.10 | Zhejiang | ORF5 | **-** | **-** | **-** | **-** | **-** | **-** | **-** |
| ZJhz16-2 | 2016.02 | Zhejiang | ORF5 | **-** | **-** | **-** | **-** | **-** | **-** | **-** |
| ZJ/HZdt/2013 | 2013.01 | Guangdong | ORF5 | **-** | **-** | **-** | **-** | **-** | **-** | **-** |
| JN1504 | 2015.04 | Jiangsu | ORF5 | **-** | **-** | **-** | **-** | **-** | **-** | **-** |
| JSYZ1803-5 | 2018.03 | Jiangsu | ORF5 | **-** | **-** | **-** | **-** | **-** | **-** | 31233656 |
| 214_HNZMD-4 | 2019.05 | Hubei | ORF5 | **-** | **-** | **-** | **-** | **-** | **-** | **-** |

Note：**-**: Lack of related data.


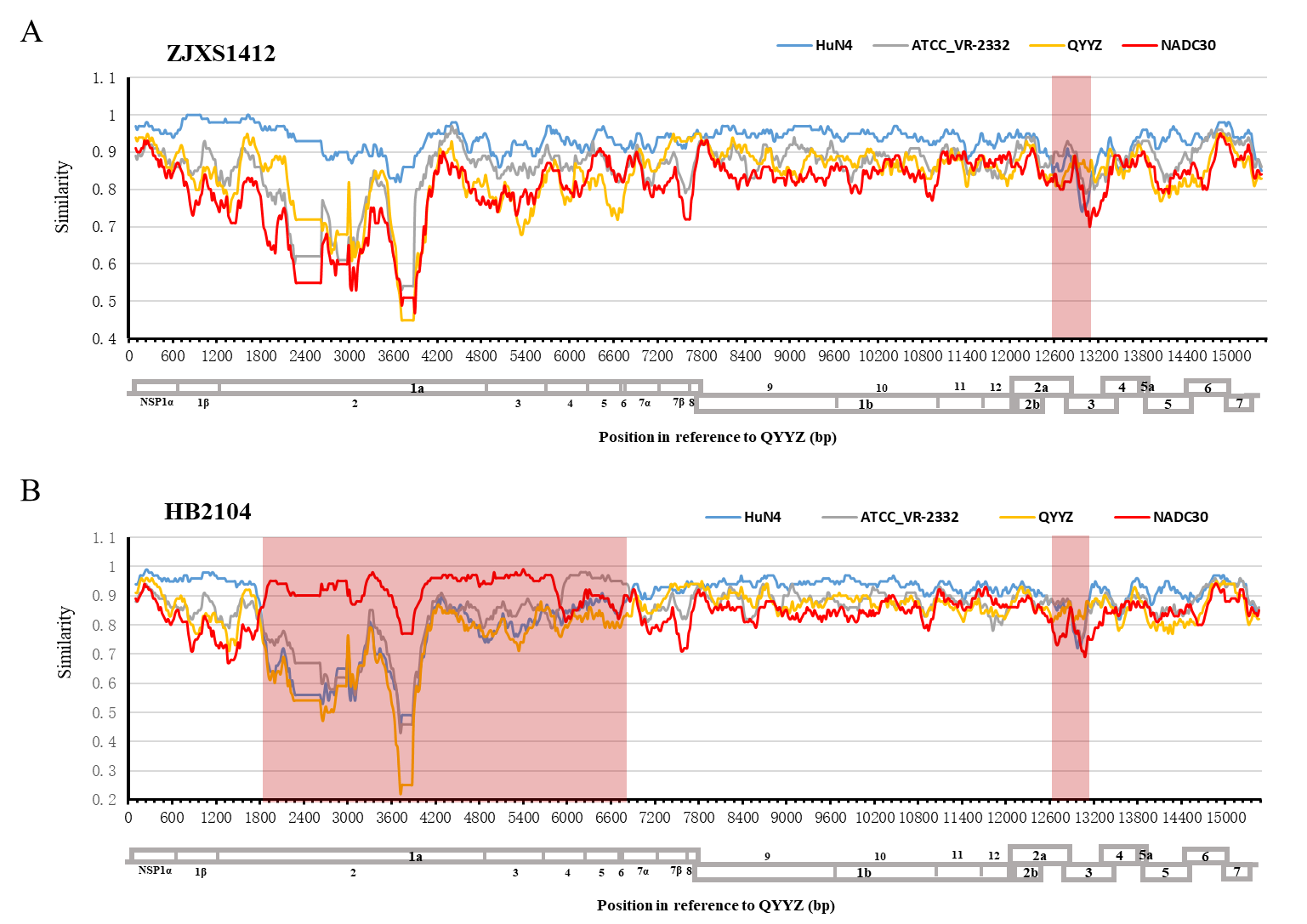


**FIGURE S1. The recombination analysis results of ZJXS1412 and HB2104.** (A) The recombination analysis results of ZJXS1412. (B) The recombination analysis results of HB2104. The red area indicates the possible recombination position
